# Supplementary material for: Association between oral microbiome and seven types of cancers in East Asian population: a two-sample Mendelian randomization analysis
Source: Front Mol Biosci. 2023 Nov 21;10:1327893. doi: 10.3389/fmolb.2023.1327893 (PMC10702768; doi:10.3389/fmolb.2023.1327893)
Supplement: Supplementary file 1 [file DataSheet1.ZIP › Supplementary Materials/MR plots of saliva/saliva═╝/ovarian cancer/pheno.2262_to_ovarian cancer_forest.pdf]

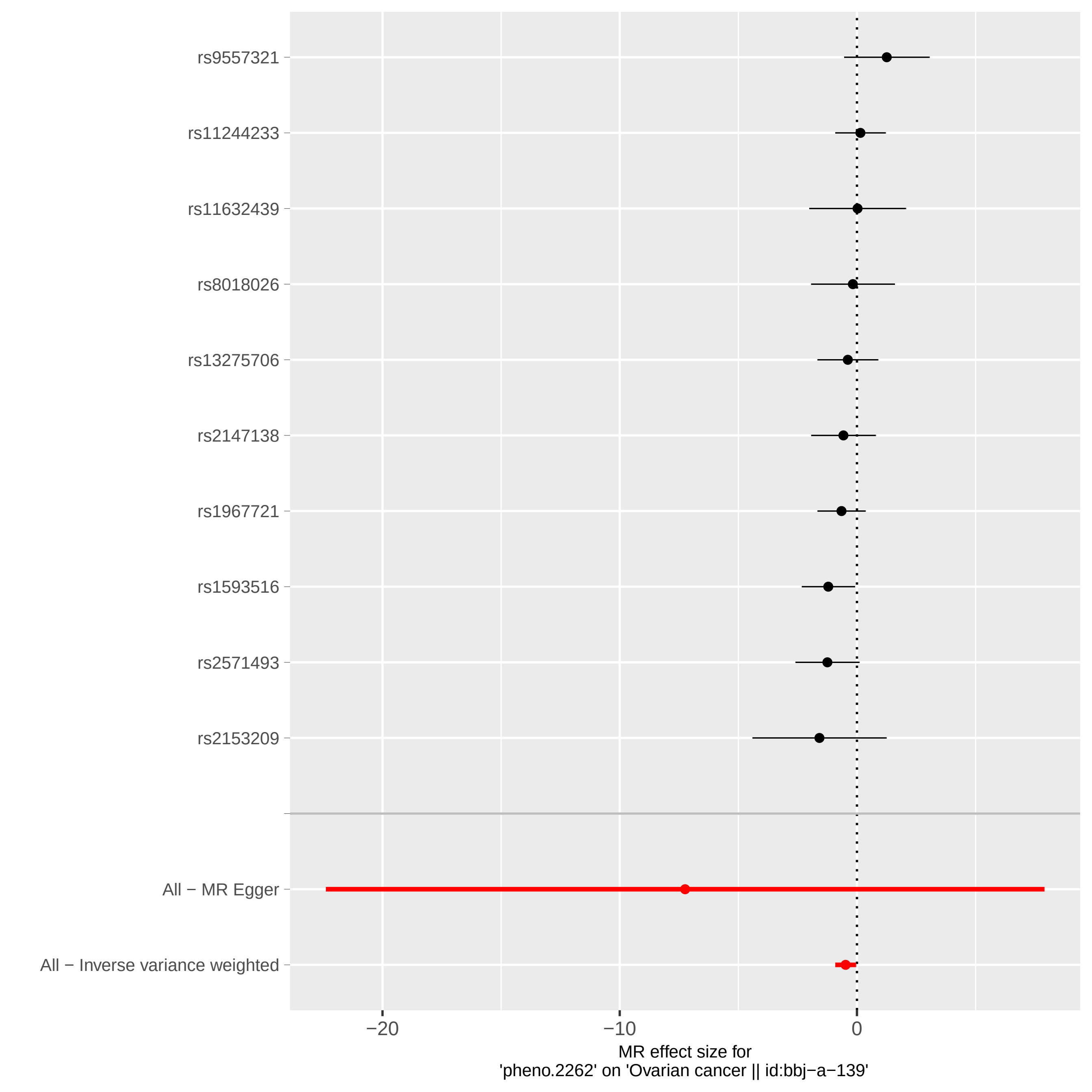

rs9557321
rs11244233
rs11632439
rs8018026
rs13275706
rs2147138
rs1967721
rs1593516
rs2571493
rs2153209
All − MR Egger
All − Inverse variance weighted
−20
−10
0
MR effect size for
'pheno.2262' on 'Ovarian cancer || id:bbj−a−139'
